# Laparoscopy in Low- and Middle-Income Countries: A Survey Study

**DOI:** 10.7759/cureus.40761

**Published:** 2023-06-21

**Authors:** Omaid Tanoli, Hamza Ahmad, Haider Khan, Awais Khan, Zoha Aftab, Mashal I Khan, Etienne St-Louis, Tanya Chen, Kathryn LaRusso

**Affiliations:** 1 Surgery, University of Toronto, Toronto, CAN; 2 Experimental Surgery, McGill University, Montreal, CAN; 3 Department of Surgery, Bacha Khan Medical College, Mardan, PAK; 4 Surgery, Hayatabad Medical Complex Peshawar, Peshawar, PAK; 5 Department of Surgery, Nottingham University Hospitals, Nottingham, GBR; 6 Internal Medicine, Khyber Girls Medical College, Peshawar, PAK; 7 Surgery, Centre for Global Surgery, McGill University, Montreal, CAN; 8 Otolaryngology - Head and Neck Surgery, University of Toronto, Toronto, CAN

**Keywords:** survey study, laparoscopy programs in lmic, laparoscopy in lmics, global surgery, : laparoscopy

## Abstract

Introduction: An increasing shift towards non-communicable diseases and an existing high surgical burden of disease in low-middle-income countries (LMICs) has impelled the need for implementing laparoscopic surgery, a safe and cost-effective surgical service. However, despite countless benefits, laparoscopic surgery programs remain limited throughout LMICs, and limited understanding is known of healthcare professionals’ views regarding the implementation of laparoscopic surgery in their local healthcare environments. Therefore, the purpose of this study is to better understand the perceived challenges and barriers to implementing long-term laparoscopic surgery programs from the perspective of healthcare professionals.

Methods: Upon receiving ethical approval from the McGill University Health Center (MUHC), a nine-question survey (concerning attributes required to establish a successful laparoscopic program in LMICs and to gain insight into what surgeons from LMICs believed were the necessary next steps) was pilot tested amongst faculty members, and subsequently disseminated to healthcare professionals practicing in LMICs. Explicit consent was obtained from the participants before answering the survey.

Results: Thirty-four participants representing a total of 35 countries participated in the survey with the majority having received laparoscopic surgery training. Overall, participant responses were characterized by two major themes. Highlighted in the first theme, Laparoscopic Experience and Training Curriculum, were responses concerning current laparoscopic training and education, improved career opportunities provided by laparoscopic training, and a particular existing potential to incorporate laparoscopic surgery into the current surgical curriculum at various levels of training. Emphasized in the second theme, Challenges and Next Steps, were responses concerning barriers to the implementation of laparoscopic surgery, current institutional capabilities, and the need for improving mentorship through existing surgical societies such as the College of Surgeons of East, Central, and Southern Africa (COSECSA), West African College of Surgeons (WACS), and The Pan-African Academy of Christian Surgeons (PAACS).

Conclusions: A buy-in from the government, hospitals, staff, and industry is crucial for the long-term implementation of laparoscopic surgery in LMICs, which can only be accomplished through increased advocacy and the dissemination of the benefits of minimally invasive surgery both economically and socially.

## Introduction

Conditions requiring surgical interventions make up approximately one-third of the global burden of disease [1,2]. In fact, it is estimated that approximately five billion people do not have readily available access to surgical services, the vast majority of whom inhabit low- and middle-income countries (LMICs) [3]. To that end, the safe and cost-effective provision of surgical services must become a priority for policymakers throughout the world. Part of surgical capacity building includes improving upon current surgical standards, with the goal to provide excellent surgical outcomes while ensuring patients do not suffer from catastrophic expenditure, harm, or sub-par treatment. As such, with the many benefits of laparoscopic surgery, a concerted effort should be taken to improve the laparoscopic surgical capabilities in LMICs.

Since its first introduction by Hans Christian Jacobaeus in 1910, laparoscopic surgery has become a mainstay in the surgical treatment of various diseases in high-income countries (HICs) [4]. Laparoscopic surgery has various advantages over open surgery which have been described in the literature including decreased blood loss, decreased infection rates, shorter lengths of stay, improved cosmesis, and less pain and medication use [5]. In fact, laparoscopic surgery can even be safely performed in conjunction with open surgery to aid in complex cases [6]. However, laparoscopic surgery still remains unavailable for a majority of the world’s population. Given the benefits of laparoscopic surgery, namely a reduced length of stay, earlier return to work, and decreased infection rates - the potential benefit of laparoscopic surgery in LMICs is likely to exceed those in HICs.

Laparoscopic surgery is associated with multiple barriers to implementation in LMICs. Previously, these have been described as a lack of reliable gas supply, lack of ability to maintain laparoscopic equipment, costs, irregular power supplies, lack of trained personnel, and a lack of motivation from both healthcare providers and the population [7-9]. However, many laparoscopic programs have been initiated despite these challenges, and innovative methods have been used to overcome many of the issues. Yet, laparoscopic surgery programs still remain sparse throughout LMICs.

As such, we developed a survey to better understand how surgeons and trainees in LMICs viewed the challenges to implementing and maintaining laparoscopic surgery programs in their specific environments. Moreover, this insight could be used to establish and progress the necessary next steps toward improving laparoscopic surgery capabilities throughout many LMICs.

## Materials and methods

Participant recruitment

A list of surgeons/allied health professionals who had worked or currently working in LMICs was obtained from the Centre for Global Surgery at the MUHC. Using the secure REDCap distribution tool, the survey was disseminated to this list of participants via email. Explicit consent was obtained from study participants upon taking the electronic survey. The consent form included information concerning the purpose of the study, participant approval to use the data for the purpose of publication, and a choice to leave a question unanswered if the surgeons/allied health professionals wished to do so. Before the commencement of the study, ethical approval was received from the McGill University Health Centre (MUHC) Research Institutional Board (#2018-3529).

Survey tool

A survey tool was developed to determine which attributes would be required in order to establish a successful laparoscopic program in LMICs and to gain insight into what surgeons from LMICs believed were the necessary next steps. To that end, a nine-question survey was developed (Appendix 1) and pilot tested amongst randomly selected faculty members. Upon finalization in 2021, a survey was distributed to our contact list for a period of two months and participants were recruited to complete the survey. 

Data analysis

Data analysis was an ongoing process. Study data was collected and managed using the REDCap system (Vanderbilt University, Nashville, TN, USA). Quantitative data was analyzed using descriptive statistics whilst the open-ended responses were thematically analyzed. The first, second, third, and fourth authors performed an iterative analysis to reach a consensus on the conceptualization of the open-ended responses.

## Results

Thirty-four surgeons/trainees participated from twenty-five different countries. Table 1 provides a comprehensive list of the representative countries.

**Table 1:** Geographical Representation of Respondents

Continent	Country	Number (n)	Percentage (%)
Asia	Pakistan	6	17.60%
Asia	Indonesia	1	2.90%
Asia	Sri Lanka	1	2.90%
Africa	South Africa	3	8.80%
Africa	Cameroon	2	5.90%
Africa	Nigeria	1	2.90%
Africa	Tanzania	1	2.90%
Africa	Sierra Leone	1	2.90%
Africa	Kenya	1	2.90%
Africa	Benin	1	2.90%
Africa	Botswana	1	2.90%
Africa	Namibia	1	2.90%
Africa	Mozambique	1	2.90%
Africa	Ivory Coast	1	2.90%
Africa	Uganda	1	2.90%
Africa	Ethiopia	1	2.90%
South and Central America	Guyana	1	2.90%
South and Central America	El Salvador	1	2.90%
South and Central America	Guadeloupe	1	2.90%
South and Central America	Ecuador	1	2.90%
South and Central America	Peru	1	2.90%
Oceania	Papua New Guinea	1	2.90%
Oceania	Vanuatu	1	2.90%
HICs	Canada	2	5.88%
HICs	USA	1	2.90%

The participants in the survey included staff surgeons/consultants, surgical trainees/residents/registrars, a biomedical engineer, and a professor of pediatric surgery (Table 2). The majority of participants worked at tertiary or national referral hospitals, while others worked at district, regional, or provincial hospitals, and some were affiliated with NGOs or private hospitals. A portion of the participants were also affiliated with universities or academic centers, and a few worked at both private and public institutions.

**Table 2:** Participants' Roles and Affiliation

Role	Number (n)	Percentage (%)
Staff Surgeons/Consultants	27	79.40%
Surgical Trainees/Residents	5	14.70%
Biomedical Engineer	1	2.90%
Professor of Pediatric Surgery	1	2.90%

Laparoscopic experience and training curriculum

Out of the thirty-four participants in the survey, a significant number of them had received laparoscopic surgical training. Among those who received training, the majority agreed that it provided them with more training and job opportunities elsewhere, while a smaller portion had a neutral stance on the matter. However, a few participants felt that laparoscopic training did not increase their job or training prospects. The overwhelming majority of participants believed that incorporating laparoscopic surgery programs into surgical training in resource-constrained settings is highly beneficial. There were differing opinions regarding the stage at which laparoscopic surgery should be introduced in surgical training, with some suggesting starting from medical school, and most advocating for inclusion during residents' or surgical trainees' training. None of the participants felt that laparoscopic surgery training should be restricted to staff or consultant levels. These characteristics are detailed in Table 3.

**Table 3:** Participants' Work Settings

Work Setting	Number (n)	Percentage (%)
Tertiary/National Referral Hospital	23	67.60%
District/Regional/Provincial Hospital	4	11.80%
NGO/Private Hospital	2	5.90%
Affiliated with a University/Academic Center	7	20.60%
Worked at both Private and Public Institutions	5	14.70%

When participants were asked about their beliefs regarding the integration of a laparoscopic program into their affiliated hospital's training curriculum in the long term. A notable portion of participants expressed agreement, with some strongly agreeing and others simply agreeing. Conversely, a smaller number disagreed, and a few participants neither agreed nor disagreed. No participants strongly disagreed with the integration. Regarding the offering of laparoscopic programs through a designated program like a fellowship training program, participants had diverse opinions. Some strongly agreed, while others agreed or held a neutral stance. A minority disagreed, and a few participants strongly disagreed with the idea. These opinions are expressed further in Table 4.

**Table 4:** Participants' Beliefs on Integration of Laparoscopic Program

	Strongly Agree	Agree	Neither Agree nor Disagree	Disagree	Strongly Disagree	Strongly Agree
Integration in Training Curriculum	12 (35.3%)	15 (44.1%)	5 (14.7%)	4 (11.8%)	0 (0%)	Integration in Training Curriculum
Offering through Fellowship Program	7 (20.6%)	13 (38.2%)	5 (14.7%)	4 (11.8%)	3 (8.8%)	Offering through Fellowship Program

Challenges and next steps

The participants highlighted several major challenges in integrating laparoscopic surgery. These challenges, ranked in decreasing order of prevalence, included a lack of governmental policy/support for laparoscopic surgery, a shortage of basic supplies, equipment, and sterilization procedures, insufficient trained allied health staff (such as nurses and OR techs), a lack of buy-in from faculty or administration, a shortage of trained staff to oversee teaching, absence of local vendors for equipment maintenance, redeployment of laparoscopic trained staff to other facilities, unreliable power supply, and a lack of troubleshoot capabilities among staff (Table 5). Additionally, participants mentioned other challenges, such as a lack of biomedical engineering capabilities, increased training time, high cost of laparoscopic consumables, and concerns regarding OR time.

**Table 5:** Major Perceived Challenges in Integrating Laparoscopic Surgery

Rank	Challenges
1	Lack of governmental policy/support for laparoscopic surgery
2	Shortage of basic supplies, equipment, and sterilization procedures
3	Insufficient trained allied health staff (e.g., nurses, OR techs)
4	Lack of buy-in from faculty or administration
5	Shortage of trained staff to oversee teaching
6	Absence of local vendors for equipment maintenance
7	Redeployment of laparoscopic trained staff to other facilities
8	Unreliable power supply
9	Lack of troubleshoot capabilities among staff
10	Lack of biomedical engineering capabilities
11	Increased training time
12	High cost of laparoscopic consumables
13	Concerns regarding OR time

Participants expressed different perspectives on the most effective approaches to incorporate laparoscopic surgery into their training curriculums. The majority (61.8%) believed that developing laparoscopic surgery capabilities within their own institution to provide longitudinal exposure would be the best approach. In contrast, a smaller percentage (8.8%) favored electives at other institutions within the same country with laparoscopic surgery capabilities, while an equal percentage (11.8%) preferred electives at foreign institutions with such capabilities. Additionally, another group (11.8%) indicated that specific fellowships in laparoscopic surgery following the completion of surgical training would be the ideal way to integrate laparoscopy into their training curriculum. Participants also highlighted the importance of teaching laparoscopic principles to medical students, interns, and medical officers as a means for them to learn the basics before engaging in clinical practice (Table 6).

**Table 6:** Suggested Approaches for Incorporating Laparoscopic Surgery into Training Curriculums

Approaches	Participants (n)	Percentage (%)
Developing capabilities at the institution	21	61.8
Electives at other institutions within the same country	3	8.8
Electives at foreign institutions	4	11.8
Specific fellowships in laparoscopic surgery	4	11.8

Finally, the participants identified several important steps for the future. These steps, ranked in decreasing order of prevalence, included improving mentorship through established surgical societies such as the College of Surgeons of East, Central, and Southern Africa (COSECSA), West African College of Surgeons (WACS), and The Pan-African Academy of Christian Surgeons (PAACS) ongoing training initiatives through partnerships with high-income countries (HICs), funding support through national programs for hospitals, promoting local production and maintenance of equipment, fostering collaborative research efforts, and enhancing quality and safety measures. Other suggestions from participants involved increasing funding for surgical services by governmental organizations, establishing long-term partnerships with dedicated stakeholders locally and internationally, establishing skills labs, and providing access to laparoscopic training modules (Table 7).

**Table 7:** Proposed Steps Going Forward for Improving Surgical Practices

Steps Going Forward	
Improving mentorship	
Ongoing training initiatives	
Funding national programs	
Local equipment production and maintenance	
Collaborative research	
Improving quality and safety measures	

## Discussion

This study surveyed multiple healthcare professionals from across the world working in LMICs. We were able to represent a wide variation in surgical systems exemplified by the inclusion of surgeons working in South America, Asia, Africa, and Oceania. Each environment will provide different challenges towards implementing laparoscopic surgical programs, however, the emphasis of this study was to determine overarching themes and lessons that could be applied generally to LMICs. As displayed in this study and previous ones, there is an interest in developing laparoscopic surgical capabilities in LMICs [5,10,11]. This interest is not limited to healthcare personnel, as there has been an increased trend in the general population in LMICs actively seeking laparoscopic surgeries when available. Given the many benefits of laparoscopic surgery, it is imperative to understand how the challenges to implementation can be overcome.

Ninety-four percent of our participants agreed that laparoscopic surgery should be a part of their training programs, with at least 79% of participants either strongly agreed or agreed that their institution would be able to have a long-term laparoscopic surgery program. As most participants were from tertiary/referral hospitals, it is likely these institutions do have the resources necessary to perform laparoscopic surgery on a daily basis. However, a major roadblock appears to be a lack of governmental or hospital support to provide safe laparoscopic surgery as indicated by over half of the participants. This may be explained by the perceived large costs associated with minimally invasive surgery. However, as has been proven throughout the literature, laparoscopic surgery is a cost-effective intervention for both hospitals and patients [12-14]. Interestingly, laparoscopic surgery has also been shown to be cost-effective as an imaging modality [7]. Regardless of its use as a therapeutic or diagnostic modality, minimally invasive surgery requires a considerable initial investment, however, these costs become justified over the long run as operating room time and patient length of stay decrease. The other challenges most noted included a lack of basic supplies, sterilization procedures and equipment, a lack of trained staff, and a lack of administration buy-in. With the right hospital and governmental policy, these challenges can all be overcome [8]. Clearly, further advocacy is required in order to garner support for laparoscopic surgery in these regions as a cost-effective intervention.

The vast majority of participants believed that the best way to incorporate laparoscopic surgery into the training curriculum is through longitudinal exposure. As is the case in many HICs, longitudinal exposure to minimally invasive surgery allows trainees to develop and fine-tune different skills at various levels of comfort with surgical procedures. However, this can only be accomplished with a fully functional laparoscopic surgery program. Other participants noted the use of electives or fellowships at foreign institutions would be ideal for minimally invasive surgical training. Although these can be useful in the initial set-up of laparoscopic programs, this is not a long-term solution. In order to ensure the sustainability of these programs, home-grown laparoscopically trained surgeons are a necessity. As indicated by participants, mentorship and associations with HICs are important steps moving forward. These are necessary for the initial set-up of laparoscopic programs; however, local champions are an integral part of progressing these programs. Without local stakeholders, who are invested in the training of future surgeons, one cannot expect the longevity of these programs.

On a similar note, participants also believed local equipment production and maintenance were of the utmost importance. The reliance of LMICs on HICs for equipment and maintenance is not a long-term solution, although may be a necessary first step. Along with governmental and hospital support, local businesses and entrepreneurs must be invested in the long-term success of these programs. There have been various examples of ingenuity and novelty with regard to the low-cost and highly effective use of resources to establish laparoscopic capabilities throughout LMICs. Gasless laparoscopy has been used to minimize the reliance on carbon dioxide insufflators [15]. Low-cost minimally invasive systems and camera systems have also been previously described [16]. Even cost-effective training models have been evaluated to minimize the costs associated with minimally invasive surgery [17]. These highlight the belief that if there is the right motivation, laparoscopic surgery can become a stable part of most healthcare systems in LMICs.

The inherent limitation of this study is that it was a survey study and is subject to the biases involved such as nonresponse bias and information bias. However, the major strength of this study is the inclusion of personnel from a myriad of working environments, allowing for the development of an overarching theme and patterns with respect to laparoscopic surgery programs.

## Conclusions

As highlighted from the results of this survey study, the key to implementing long-term laparoscopic surgery programs in LMICs is buy-in from the government, hospitals, staff, and industry. This can only be accomplished through increased advocacy and the dissemination of the benefits of minimally invasive surgery both economically and socially. As seen with the recent COVID-19 pandemic, hospital resources are scarce and inefficiencies in hospital systems have been highlighted. Moving forward, healthcare policy should focus on the efficient use of healthcare resources including operating rooms, hospital beds, and funds. In the current climate, the implementation of minimally invasive surgery should be seen as a necessity rather than a luxury, and work towards this goal is of the utmost importance.

## Figures and Tables

**Figure 1:**
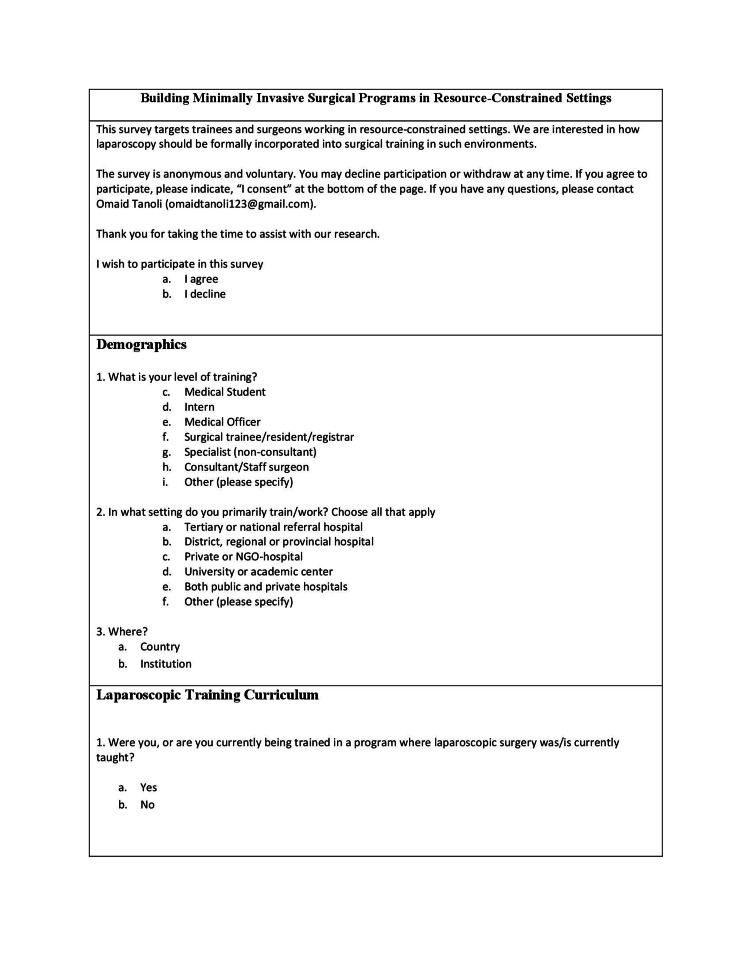
Building Minimally Invasive Surgical Programs in Resource-Constrained Settings Survey

**Figure 2:**
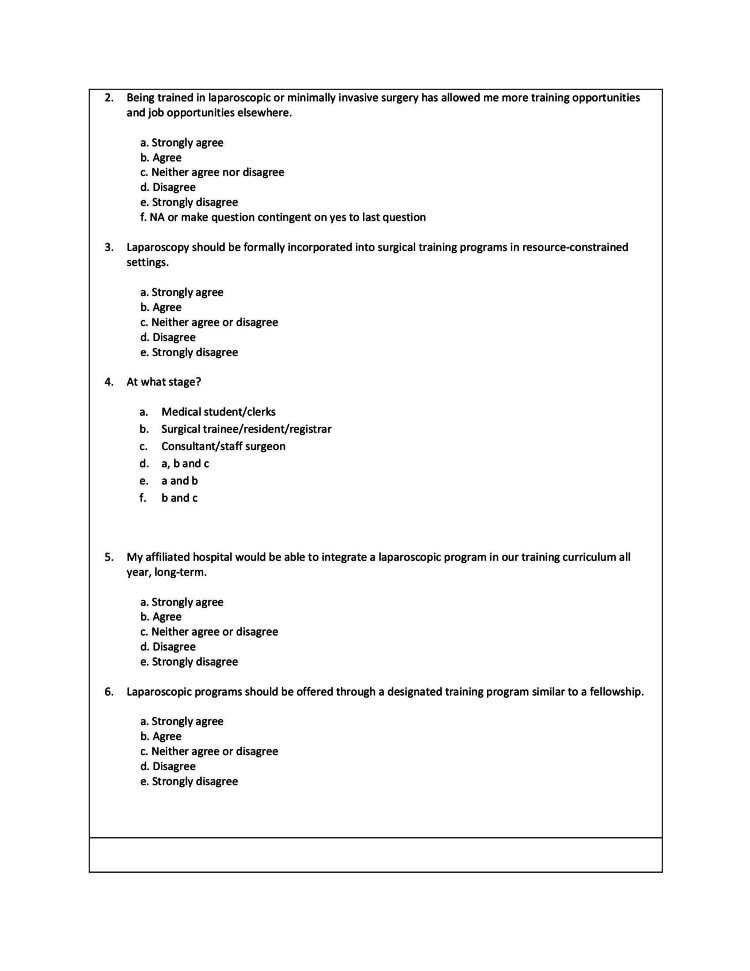
Building Minimally Invasive Surgical Programs in Resource-Constrained Settings Survey

**Figure 3:**
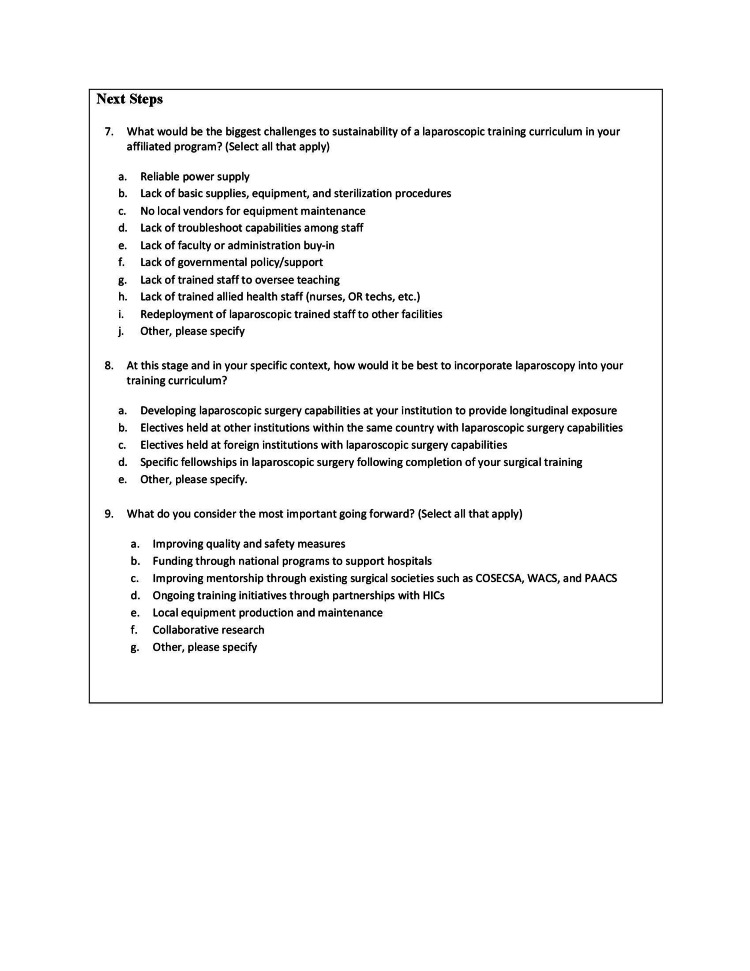
Building Minimally Invasive Surgical Programs in Resource-Constrained Settings Survey
